# Pea-Protein-Stabilized Emulsion as a High-Performance Cryoprotectant in Frozen Dough: Effects on the Storage Stability and Baking Performance

**DOI:** 10.3390/foods13233840

**Published:** 2024-11-28

**Authors:** Diming Li, Youqing Shi, Zhihan Ouyang, Yongxin Teng, Boru Chen, Yingying Chen, Yufan Luo, Nan Zhang, Nandan Kumar, Yonghui Li, Bin Li, Xiangwei Zhu

**Affiliations:** 1 Guangdong Key Laboratory of Intelligent Food Manufacturing, Foshan University, Foshan 528225, Chinabrchen@fosu.edu.cn (B.C.); 2 Key Laboratory of Fermentation Engineering (Ministry of Education), Hubei Key Laboratory of Industrial Microbiology, Cooperative Innovation Center of Industrial Fermentation (Ministry of Education & Hubei Province), National “111” Center for Cellular Regulation and Molecular Pharmaceutics, Hubei University of Technology, Wuhan 430068, China; 3 Department of Grain Science and Industry, Kansas State University, Manhattan, KS 66506, USAnandan@ksu.edu (N.K.); yonghui@ksu.edu (Y.L.); 4 College of Food Science and Technology, Huazhong Agricultural University, Wuhan 430070, China; libinfood@mail.hzau.edu.cn

**Keywords:** pea protein, oil-in-water emulsion, frozen dough, storage stability, baking performance

## Abstract

The use of oil-in-water (O/W) emulsion has drawn increasing attention in the baking industry. Compared with some of the well-recognized functionalities, such as textural improvers and flavor carriers, its cryoprotective behavior in frozen dough has not been extensively investigated. Herein, this study reported a pea-protein (PP)-stabilized O/W emulsion with good freeze–thaw stability and evaluated its effectiveness as a high-performance dough cryoprotectant. Specifically, the emulsions were stabilized by 2, 3, and 4 wt% of PP (PP-2, -3, and -4, respectively) and incorporated into frozen doughs, whose cryoprotective effects were systematically evaluated in terms of dough storage stability and baking performance after 4 weeks of storage. Results showed that the frozen dough with PP-3 emulsion exhibited the most uniform water distribution and reduced content of freezable water as reflected by the results from differential scanning calorimetry and low-field nuclear magnetic resonance analyses. Moreover, the PP emulsion helped to maintain the integrity of the gluten network, thus enhancing the dough elasticity. Accordingly, the emulsion-added bread samples exhibited significantly improved loaf volume and textural properties (e.g., softness) and less baking loss. Our findings highlighted the potential of PP emulsion as a viable and high-performance dough cryoprotectant.

## 1. Introduction

The global market demand for freezer-to-oven products has substantially grown due to a faster-paced lifestyle and the need for standardization of on-site food preparation. By 2026, the global baked products market is projected to reach USD 457.4 billion, reflecting this upward trend [1]. The increased demand has prompted great interest in frozen dough technology among manufacturers and retailers who aim to offer high-quality products to consumers [2]. However, frozen dough formulation still faces critical challenges related to long-term storage capability, particularly moisture loss and texture degradation issues, especially in high-fat formulas; concerns of dough hardening and palatability loss are common with frozen doughs [3]. Therefore, effective cryoprotective strategies are critical in formulation development to optimally retain the fresh state of dough throughout frozen storage and thus the consistency in the final baked products.

Cryoprotectants, in the form of dough conditioners, have been used as additives to reduce ice crystal formation during freezing. Previous studies have explored the use of polysaccharides, phosphates, and protein hydrolysates as cryoprotectants in dough products [4,5]. These additives, such as carboxymethyl cellulose and chickpea protein hydrolysates, have been shown to inhibit ice recrystallization and protect the secondary and tertiary structures of gluten proteins, thus preserving the dough viscoelastic properties [6,7]. The effectiveness of cryoprotectants is frequently dosage-dependent [8]. However, excessive cryoprotectant usage raises concerns regarding both economic feasibility in manufacturing and the possibility that it may alter the overall taste. Therefore, there is an urgent need to develop a formulation strategy to reduce the use of additives while retaining cryoprotection effectiveness.

Lipophilic compounds, such as butter and vegetable shortenings, are frequently utilized in dough products as flavoring and crumb texturizing ingredients [9]. Since flour dough is largely hydrophilic, lipid materials require emulsifiers to achieve uniform distribution in the dough [10]. Common emulsifiers include egg protein and soy protein, along with synthetic options like mono- and di-glyceride and sodium stearoyl lactylate [11]. The use of oil-in-water (O/W) emulsions is a promising approach to achieve the dual functions of lipid incorporation and improved cryostability. By lowering the freezing point, preventing the formation of ice crystals, and increasing material viscosity, O/W emulsions can lessen dehydration and water migration [12]. Despite these potential advantages, the use of O/W emulsions has primarily been limited to fresh dough. For example, Bu et al. found that an O/W emulsion made with octenyl succinic anhydride (OSA) and konjac glucomannan (KGM) improved the texture of cakes, enhancing their adhesiveness, elasticity, hardness, and chewiness [13]. Similarly, Sanz et al. used a sunflower seed oil–water–cellulose ether emulsion in biscuit dough and produced a crisper texture in the final baked product [14]. The cryoprotective properties of emulsion in frozen dough have not been extensively studied. This limitation arises from the concern that O/W emulsions are prone to destabilization during freeze–thaw (FT) cycles, which can lead to phase separation, emulsifier flocculation, and reduced cryoprotective performance [15]. Therefore, selecting the appropriate emulsifiers is critical for maintaining water–oil interface stability at low temperatures when developing high-performance emulsion cryoprotectants for frozen dough products.

Plant proteins have garnered significant attention when screening the appropriate emulsifiers because plant proteins have lower thermal hysteresis activity and higher ice recrystallization inhibition than fish and insect proteins [16]. Among them, soy protein is a commonly used plant-based emulsifier. Specific soy protein isolates with sedimentation coefficients of 7 S and 11 S are frequently added to various products, including infant formula, due to their functional and nutritional benefits [17]. However, soy protein is also a known allergen [18], which underscores the need to explore alternative plant proteins suitable for oil-in-water emulsions in frozen dough applications, aligning with economic and functional efficiency [19,20]. Recently, we reported that pea-protein-based O/W emulsions prepared via ultrasonication demonstrated excellent freeze–thaw stability [21]. Additionally, pea protein, with its favorable amino acid profile and hypoallergenic properties, has garnered interest as a baking additive. Like other pulse proteins, it also offers a clean-label alternative and has shown promise as a functional ingredient in dough products.

Based on these advantages, this study aimed to develop an emulsion system based on pea proteins that would improve the baking and storage stabilities of frozen dough by acting as a high-performance cryoprotectant. Specifically, the PP-stabilized O/W emulsions were prepared with varying protein concentrations and added into dough before freezing. The cryoprotective performances were evaluated by examining water states, rheological properties, and the microstructure of the doughs over a four-week storage period at −18 °C. Furthermore, the baking performances of re-equilibrated dough, including textural properties, specific loaf volumes, and baking losses, were also assessed. By investigating the cryoprotective effects of PP-based emulsions in frozen dough, this study provides novel insights and a scientific basis for dough cryopreservation.

## 2. Materials and Methods

### 2.1. Materials

Crude pea protein powder (80% protein content) was obtained from Baichuan Kangze Biotechnology Co., Ltd. in Xianyang, China. Wheat flour was sourced from Xinxiang Grain and Oil Processing Co., Ltd. in Xinxiang, China. Yeast and butter were supplied by Angel Yeast Co., Ltd. in Yichang, China. Additional chemical reagents, including 5,5′-dithiobis-(2-nitrobenzoic acid) (DTNB), ethylenediaminetetraacetic acid (EDTA), and hydrochloric acid (HCl), were of analytical grade and acquired from Sinopharm Chemical Reagent Co., Ltd. in Shanghai, China. Ultrapure water, with a conductivity of 0.06 µS cm^−1^, was utilized in whole experiments.

### 2.2. Preparation of Pea Protein (PP) Emulsion

#### 2.2.1. Preparation of PP

Pea protein (PP) was prepared following our previous methods with slight modifications [1]. The crude pea protein powder was mixed with ultrapure water at a 1:10 ratio (*w*/*v*), and the pH was adjusted to 9.0. The mixture was stirred for 3 h (RO 10, IKA Instruments Ltd. in Staufen, Germany) and then centrifuged at 8000 rpm (radius of 7.5 cm) for 20 min at 4 °C (HITACHI CR21N, Hitachi Manufacturing Institute in Tokyo, Japan) to obtain the supernatant. The pH of the supernatant was set to 4.5 and stood for 12 h, followed by centrifugating at 10,000 rpm (radius of 7.5 cm) for 20 min at 4 °C to collect the precipitate. The precipitate was washed three times with ultrapure water, dissolved in deionized water at a 1:5 (*w*/*v*) ratio, and adjusted to pH 7.0. The solution was then dialyzed for 48 h and freeze-dried to yield the final PP product.

#### 2.2.2. Preparation of PP Emulsion

The formulations of the PP-based emulsion are detailed in Table 1. Butter was melted in a water bath at 70 °C. The PP content was set at 2% (1.4 g), 3% (2.1 g), and 4% (2.8 g) of the total weight of water and butter (70 g), designated as PP-0, PP-2, PP-3, and PP-4, respectively, with PP-0 serving as the control. PP powder was dissolved in 65 g of ultrapure water and stirred for 30 min. Subsequently, the PP solution and butter were mixed at a mass ratio of 13:1. The mixture was homogenized at 12,000 rpm (radius of 7.5 cm) for 2 min through a high-speed homogenizer (T18, IKA Instruments Ltd., Germany) [21].

### 2.3. Determination of Droplet Size of PP Emulsion

The emulsion droplet size was measured by a Mastersizer 2000 (Malvern Instruments Ltd. in Worcestershire, UK) [22]. Both fresh and frozen emulsion samples were dispersed in deionized water for measurement with a shading rate at 10%. The refractive indices of the dispersed phase and the dispersion medium (ultrapure water) were 1.48 and 1.33, respectively.

### 2.4. Preparation of Frozen Dough

Wheat flour, sugar, salt, yeast, and the prepared emulsion were combined using a dough mixer (HM740, Hanshang Co., Ltd. in Qingdao, China) at a weight ratio of 100:10:1:2:70 and kneaded for 30 min. The leavened dough was then divided into spherical portions weighing 2.0 ± 0.1 g, 5.0 ± 0.1 g, and 40.0 ± 0.1 g. These dough portions were packed in polyethylene bags, conditioned at −30 °C for 2 h, and subsequently transferred to a refrigerator at −18 °C for storage over a period of 4 weeks. Dough samples from each group were taken at different storage intervals (0, 1, 2, and 4 weeks), thawed at room temperature (25 ± 0.5 °C) for 1 h, and then subjected to further characterization.

### 2.5. Determinationof Frozen Dough

#### 2.5.1. Freezable Water Content of Frozen Dough

Differential scanning calorimetry (DSC1, Mettler-Toledo, Zurich, Switzerland) was used to determine the amount of freezable water in frozen dough, slightly altering the Baier-Schenk method [23].

#### 2.5.2. Low-Field Nuclear Magnetic Resonance (LF-NMR)

A low-resolution NMI20-015V-1 NMR spectrometer (Niumai Electronic Technology Co., Ltd., Shanghai, China) was used to assess transverse relaxation time (*T*_2_) analyses. To conduct the Carr–Purcell–Meiboom–Gill (CPMG) test, the thawed dough samples (5.0 ± 0.1 g) were placed in nuclear magnetic test tubes. And the parameters were followed: echo time = 0.25 ms, number of echoes = 1600, number of scans = 4, and successive scan = 400 MS [24]. The *T*_2_-fit algorithm was utilized to fit the obtained CPMG pulse sequences. Additionally, using magnetic resonance imaging (MRI) with an echo time of 20 ms, a successive scan time of 800 ms, and scan repetitions of 256, the water distributions in frozen dough were discovered.

#### 2.5.3. Rheological Tests

In accordance with our earlier procedure, the rheological characteristics of thawed dough samples were assessed using a stress-controlled rheometer (MCR-92, Anton Paar, Graz, Austria) [7]. The 1.0 ± 0.2 g dough samples were loaded between two parallel plates (diameter: 25 mm, gap: 1 mm) after being thawed for 40 min at 25 °C. A tiny bit of silicone oil was put to the samples’ edge to stop moisture loss. A frequency sweep test was conducted between 0.1 and 80 Hz. Every test was conducted inside the linear viscoelastic zone at 25 °C with a constant strain of 0.5%. To evaluate changes in the rheological behaviors of the dough, the storage modulus (*G*′), the loss modulus (*G*″), and the values of tan *δ* (*G*″/*G*′ at a frequency of 1 Hz) were recorded.

#### 2.5.4. Scanning Electron Microscope

After 0, 1, 2, and 4 weeks, the microstructures of the frozen dough were examined using a JSM-6390 LV scanning electron microscope (SEM) (JEOL, Tokyo, Japan). The 2 × 2 × 2 mm dough samples were lyophilized, frozen in liquid nitrogen, then attached to the sample stage with conductive glue. The samples were then sputter-coated with gold and examined at 1000× magnification using an accelerating voltage of 20 kV [25].

#### 2.5.5. Contents of Sulfhydryl Groups

In accordance with an earlier technique, Ellman’s reagent was used to measure the amount of the sulfhydryl group in the dough [26]. To create fine particles, the lyophilized dough was crushed with a mortar and pestle and then run through a 60-mesh sieve with a 0.2 mm opening. For one hour, 0.1 g of dough powder was mixed with 10 mL of Tris–HCl buffer (80 mmol/L Tris, 4.0 mmol/L EDTA, 90 mmol/L glycine, pH 8.0) and vortexed every fifteen minutes. Following a 20-min centrifugation at 10,000 rpm (radius of 7.5 cm), 1 mL of Ellman’s reagent was added to the supernatant. A UV spectrometer (UV-1700, Shimadzu, Japan) was used to measure the solution’s absorbance at 412 nm after an additional half-hour incubation at 25 °C. The level of sulfhydryl groups was converted to micromoles per gram of lyophilized sample (μmoL/g) through a standard curve prepared from cysteine.

### 2.6. Preparation and Characterization of Bread

#### 2.6.1. Preparation of Bread

The frozen doughs were thawed at 4 °C for 12 h in a refrigerator, then transferred to a fermentation room maintained at 30 °C with 85% humidity for 75 min. Following fermentation, the doughs were molded and baked at 180 °C for 15 min. After baking, the breads were allowed to cool at room temperature (25 ± 0.5 °C) for 1 h before testing.

#### 2.6.2. Bread Specific Volume and Baking Loss

The baking loss was determined by measuring the mass difference before and after baking (*M*_1_ and *M*_2_) using the following equation [27]:

(1) $$\mathrm{Baking}\;\mathrm{loss}\;(\%)=\frac{M_{1}-M_{2}}{M_{1}}\times 100$$

The baked bread after 1 h of cooling was weighed (*M*_2_); their volumes were determined by the millet replacement method, and the specific volume (*V*) was calculated by dividing the volume by weight [28]. The specific volume of the dough was calculated using the following equation:

(2) $$\mathrm{Specific}\;\mathrm{volume}\;\left(\mathrm{mL}/g\right)=\frac{V}{M_{2}}$$

#### 2.6.3. Color Analysis

The bread was cut into 25 mm slices, and the colors of the crust and the crumb were measured using a tristimulus color analyzer (YS3020, Shenzhen Threenh Technology Co., Ltd. in Shenzhen, China).

#### 2.6.4. Textual Analysis

##### Porosity of the Bread

According to the method of Gao et al. [29], 25 mm thick slices of bread were prepared. Each slice was scanned at 600 dpi using a flatbed scanner (CanoScan 9000F Mark II, Canon Inc. in Tokyo, Japan), and the resulting black-and-white images were saved. A 40 × 40 mm area near the crumb was then cropped from each image. These cropped images were transformed into binary images using the Otsu thresholding method by Image J (1.46r, National Institute of Health in Bethesda, USA). They were subsequently exported to Image Pro Plus (version 7, Media Cybernetics in Rockville, USA) to measure the porosity.

##### TPA Analysis

The bread was cut into 2 × 2 × 2 cm cubes. Then, texture profile analysis (TPA) was used to measure the textural properties of bread by a TA.XT. Plus texture analyzer (Stable Micro Systems in Godalming, UK) equipped with a P/36R probe [30]. The measurements were conducted with pre-test and post-test speeds set at 5.0 mm/s, while a test speed of 1 mm/s was used. The trigger force was set at 5 g, and the deformation level was set at 50% of the sample height. Beginning in the initial position, the probe moved toward the test sample at a pre-measured speed before deforming it at the testing speed. After achieving the required degree of distortion, the probe returned to the compression trigger point for five seconds [31]. Finally, the probe returned to the location prior after compressing the same deformation again. The qualities of hardness, gumminess, chewiness, resilience, cohesion, and springiness were the main indications reached by this investigation [32].

##### Crumbliness Analysis

According to the method of Purhagen et al. [33], the crumbliness of the bread was measured. The crumbs after repeated slicing were collected and weighed. Each bread was cut into 10 slices, and the crumbliness was expressed as Δ g (the weight of crumb)/10 slices.

#### 2.6.5. Sensory Evaluation

According to the method of Cacak-Pirtezak et al. [34], sensory evaluations were conducted within 24 h after baking, assessing four sensory attributes: visual, touch, smell, and taste. The evaluations were conducted by a team of 20 panelists from the Philips Hydrocolloid Laboratory at Hubei University of Technology, aged 20 to 45. Each bread sample was rated on a 10-point hedonic scale, where 1 represented “very undesirable” and 10 indicated “very favorable”.

### 2.7. Statistical Analysis

All experiments were independently conducted in triplicate. One-way ANOVA was used to examine the data, followed by Duncan’s multiple comparison test, with SPSS 26.0 software (SPSS Inc., Chicago, IL, USA), and the results were presented as mean ± standard deviation. Sensory evaluation data were analyzed using the nonparametric Kruskal–Wallis test and displayed as median ± quartile. All statistical significance was set at *p* < 0.05.

## 3. Results and Discussion

### 3.1. Freeze–Thaw Stability of PP Emulsions

**Figure 1:**
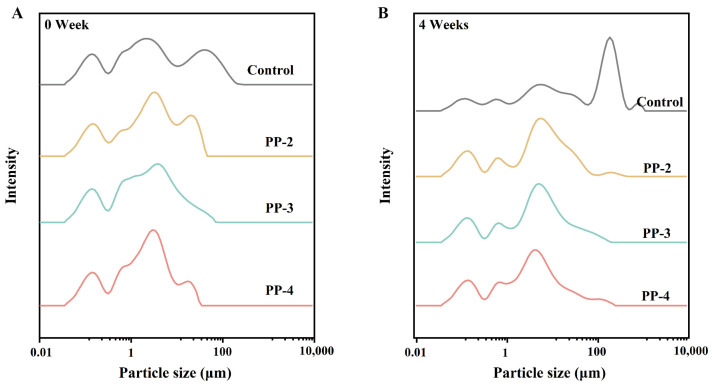
Particle size characteristics of PP emulsion. (**A**) Particle size distributions of fresh emulsion. (**B**) Particle size distributions of emulsion after 4 weeks of frozen storage.

**Figure A1:**
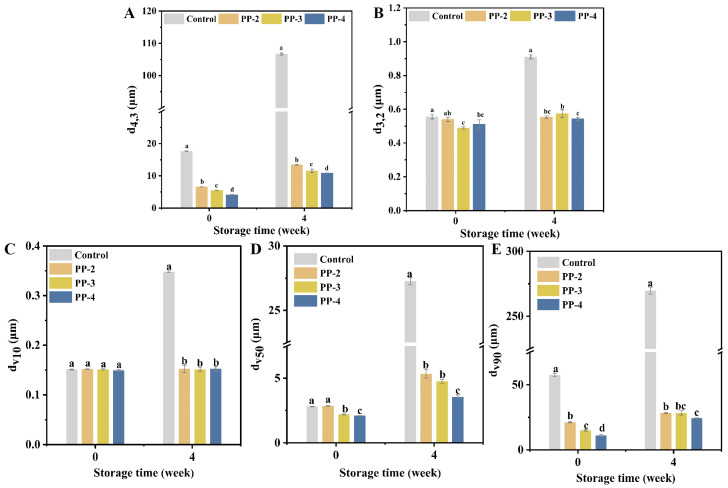
Particle size characteristics of PP emulsion: (**A**) *d*_4,3_, (**B**) *d*_3,2_, (**C**) *d*_v10_, (**D**) *d*_v50_, and (**E**) *d*_v90_. A statistically significant difference between samples with various PP emulsions at the same storage time is shown by different lowercase letters (*p* < 0.05).

Particle size is often used as an important indicator to evaluate the freeze–thaw stability of emulsions [35]. As shown in Figure 1A,B, the particle size distribution of the control group exhibited a multimodal distribution, characterized by larger emulsion droplet size and increased droplet aggregation after frozen storage. To capture all significant peaks in the distribution, *d*_4,3_, *d*_3,2_, *d*_v10_, *d*_v50_, and *d*_v90_ were measured as shown in Figure A1. After frozen storage, the *d*_4,3_ value for the control group significantly increased from 17.62 μm to 106.67 μm, showing the largest observed increase (Figure A1C). In contrast, with increasing PP concentration, the increment in *d*_4,3_ values was relatively smaller, ranging from 6.055 μm to 6.821 μm. This trend was also observed in *d*_3,2_, likely due to enhanced interfacial strength among PP molecules, which improved surface hydrophobicity, emulsifying activity, and interfacial adsorption. These enhancements promoted droplet dispersion, reduced the particle size, and bolstered the freeze–thaw stability of the emulsion [21].

Significant differences were observed in *d*_v50_ (Figure A1D) and *d*_v90_ (Figure A1E) at 0 week, with the exception of *d*_v10_ (Figure A1C). The smaller particle size, especially in *d*_v90_, might indicate that PP had excellent emulsifying characteristics. After 4 weeks, *d*_v10_, *d*_v50_, and *d*_v90_ of the control group increased by about 0.2 μm, 24 μm, and 210 μm, respectively, likely due to emulsion aggregation after freezing. In the experimental groups, the values of *d*_v10_ remained stable even after freezing, suggesting that *d*_v10_ did not have high concentration dependence on PP. However, with the increase in the PP concentration, stronger inhibitory effects on emulsion aggregation were observed in *d*_v50_ and *d*_v90_ (particularly *d*_v90_), indicating that the emulsion retained considerable stability after freezing. These results suggest that PP emulsion had strong potential as an effective cryoprotectant.

### 3.2. Moisture State and Rheological Behavior of Frozen Dough

#### 3.2.1. Freezable Water Content

**Figure 2:**
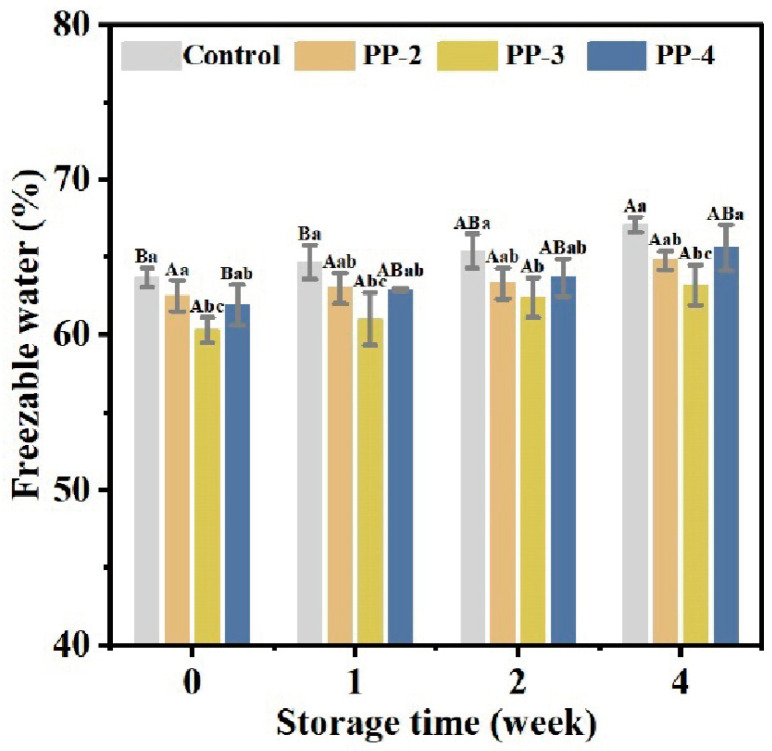
Effect of PP emulsion on freezable water in dough during 4 weeks of frozen storage. A statistically significant difference between samples of the same PP emulsion at different storage times is shown by different uppercase letters (*p* < 0.05). A statistically significant difference between samples with various PP emulsions at the same storage time is shown by different lowercase letters (*p* < 0.05).

Freezable water is a crucial factor influencing dough quality. Upon freezing, freezable water transforms into ice crystals, whose formation and expansion can disrupt the structure of the gluten network, leading to reduced elasticity and ductility of the gluten [26,36]. Generally, the content of freezing water increases with the prolonged storage time. In the control group, the freezable water content rose from 63.68% to 67.05% over 4 weeks (Figure 2). In contrast, following four weeks of storage, the freezable water contents for the PP-2, PP-3, and PP-4 groups were measured to be 64.77%, 63.17%, and 65.56%, respectively, which were obviously lower than that of the control group. This indicated that the PP emulsion mitigated the increase in the freezable water content during frozen storage. In contrast to PP emulsion, commercial emulsifiers like polysorbate 80, plant shortening, and calcium stearoyl-2-lactylate, as reported by Matuda et al. [37], showed no significant changes in the freezable water content. This suggests that stronger hydrophilic properties of PP emulsion enhance water binding, thereby reducing freezable water and limiting ice crystal growth [38].

#### 3.2.2. Water Migration and Distribution in the Frozen Dough

**Figure 3:**
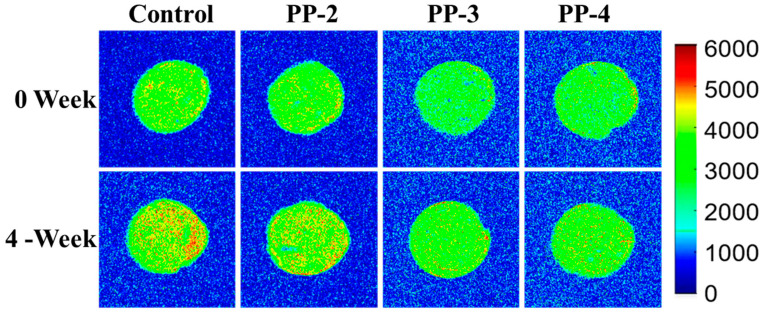
Proton density distributions of fresh (0 week) and frozen (4 weeks) dough prepared from different PP formulations.

Furthermore, LF-NMR was utilized to assess water mobility levels in frozen dough samples (PP-2, -3, -4, and control group) by analyzing *T*_2_ relaxation times and populations over various storage periods [39]. The examination of dough images was conducted to evaluate the spatial distribution and migration of water molecules by observing hydrogen proton density [40]. In Figure 3, the results depicted uniform signal intensities of hydrogen protons across all groups at week 0, indicating a homogeneous distribution of water molecules. After 4 weeks of frozen storage, the emergence of red and/or yellow dots suggested an increase in the free water content.

To analyze the moisture migration pattern in the dough during the freezing process, the water states in the dough were classified by relaxation time into three categories: (a) bound water (*T*_21_: 0.01–3.05 ms), (b) immobilized water (*T*_22_: 3.05–75 ms), and (c) free water (*T*_23_: 75–500 ms) [38]. As shown in Table 2, the proportions of *T*_21_, *T*_22_, and *T*_23_ in the fresh dough exhibited no significant difference between the control and experimental groups. However, with the extension of frozen storage time, *T*_21_ in the control group decreased from 14.71% at 0 week to 12.86% at week 4. In contrast, after 4 weeks of storage, the *T*_21_ content in dough samples with PP-2, PP-3, and PP-4 was measured at 13.85%, 14.29%, and 14.31%, respectively, significantly higher than that of the control group. These results showed that PP emulsion can effectively inhibit the transformation of the immobilized water to free water in the dough. The enhanced water-binding capacity of the dough, likely due to the incorporation of the PP emulsion, resulted in the entrapment of a greater amount of free water [41].

#### 3.2.3. Rheological Properties of the Frozen Dough

**Figure 4:**
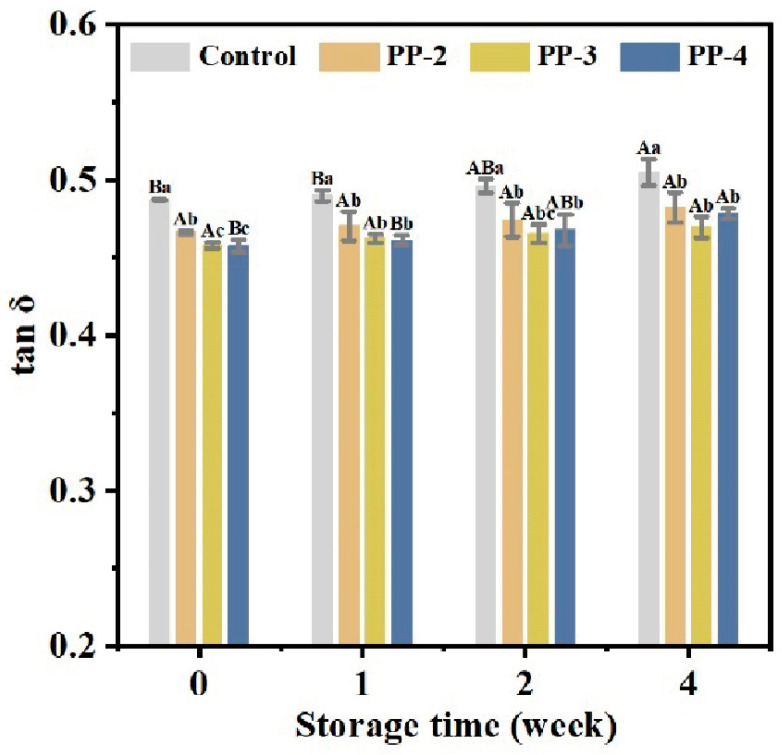
Effect of PP emulsion on rheological behavior in dough during 4 weeks of frozen storage. A statistically significant difference between samples of the same PP emulsion at different storage times is shown by different uppercase letters (*p* < 0.05). A statistically significant difference between samples with various PP emulsions at the same storage time is shown by different lowercase letters (*p* < 0.05).

The rheological behavior of the dough had a significant change as frozen storage progresses [42]. The tan *δ* values of all samples, being less than 1 (Figure 4), indicated that the elasticity was greater than the viscosity, demonstrating a solid-like state of the dough. At the initial stage (0 week), the viscoelasticity of the fresh dough exhibited considerable variation. Notably, the elastic modulus of the dough with the addition of PP emulsion significantly increased, resulting in a decrease in tan *δ*. Over the storage period, the tan *δ* value of the control group gradually increased from 0.48 to 0.51. In contrast, tan δ values in the PP groups were less affected; for instance, the tan *δ* value of the PP-3 sample only increased to 0.47. These results indicated that the incorporation of PP emulsions can effectively slow down the depolymerization of gluten aggregates and mitigate the reduction in dough elasticity associated with freezing. It is speculated that PP emulsions, characterized by high freeze–thaw stability, possess specific gelling properties that may facilitate gluten formation and cross-linking. This can strengthen the dough structure and enhance its viscoelasticity [43].

### 3.3. Microstructure and the Free Sulfhydryl Group (-SH) Content of Frozen Dough

**Figure 5:**
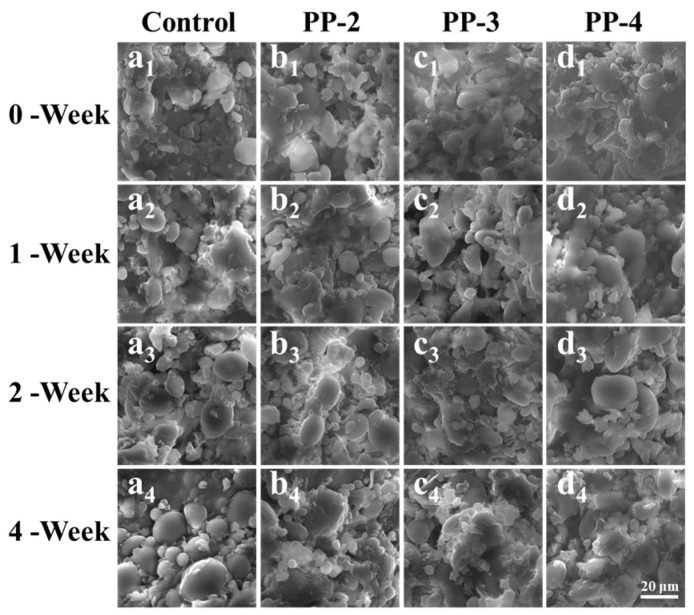
Effect of PP emulsion on dough microstructures during frozen storage (0–4 weeks). ((**a**–**d**) are control, 2% PP, 3% PP, and 4% PP, respectively).

The microstructure of the dough is shown in Figure 5, which consists primarily of a gluten network interspersed with small starch granules. The microstructure of the fresh dough (0 week) appeared relatively compact, with starch granules predominantly taking on hemispherical shapes. However, with extended freezing time, the starch particles tended to detach from the gluten protein network, resulting in the formation of more spherical structure [2]. After 4 weeks of frozen storage, the microstructure of the dough containing PP emulsion remained relatively compact, effectively filling the gaps between the gluten network and starch granules. This stability may be attributed to the influence of PP emulsion on the hydration of proteins in the dough. Furthermore, the PP emulsion might interact with the proteins of flour to advance the formation of a gluten network structure [44].

**Figure A2:**
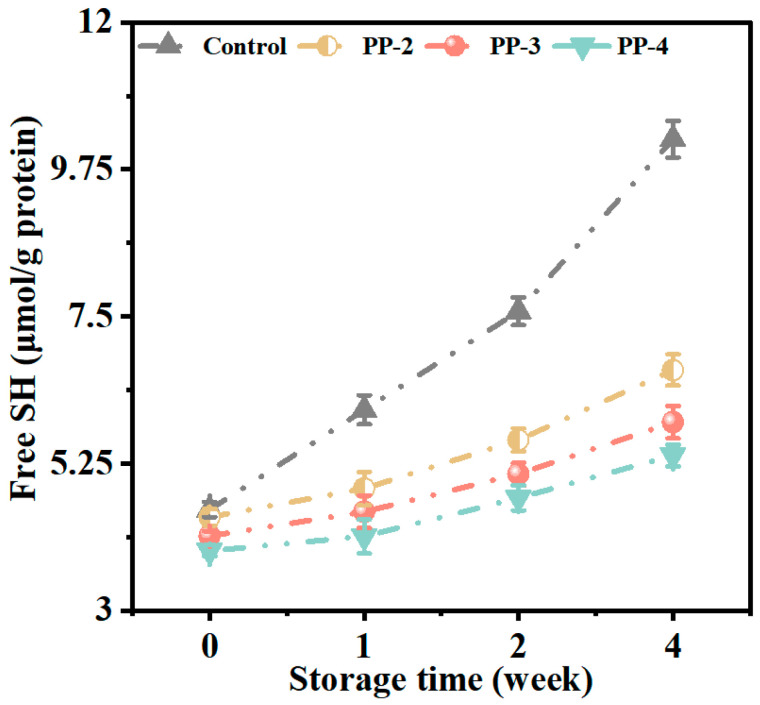
Effect of PP emulsion on sulfhydryl content in dough during frozen storage (0–4 weeks).

During prolonged storage of frozen dough, the degradation of disulfide bonds within the gluten matrix constitutes the primary structural deterioration mechanism [45]. The changes in free sulfhydryl levels of gluten were assessed across various frozen dough samples (Figure A2). In the control group, the content of free sulfhydryl groups significantly increased from 4.54 to 10.21 μmol/g after 4 weeks of storage, indicating a substantial degradation of disulfide bonds within the gluten. This increase was likely due to recrystallization processes occurring during frozen storage, which inflict mechanical damage on the gluten structure [43]. Conversely, the PP groups exhibited reduced cleavage of gluten disulfide bonds, as evidenced by the lower concentrations of free sulfhydryl groups, i.e., 6.67 μmol/g for PP-2, 5.87 μmol/g for PP-3, and 5.37 μmol/g for PP-4 after 4 weeks. Changes in free SH levels served as a significant indicator of alternations in S–S bonds [46], which were crucial for maintaining a three-dimensional gluten network. This finding aligned with the results from the SEM, which revealed a continuous gluten network. The reduction in free sulfhydryl groups might play a role in preventing ice crystals from damaging the dough structure.

### 3.4. Bread Characteristics

Based on the above measurements, the protective effect of PP emulsion on frozen dough has been confirmed through multiple indicators, including reductions in freezable water content, moisture distribution, protein state, and microstructure of the dough. To gain deeper insights into the baking performance of the dough containing PP emulsion, further evaluations were conducted, measuring baking loss, specific volume (v/m), color, texture properties (porosity, hardness, gumminess, chewiness, resilience, cohesiveness, springiness, and crumbliness), and sensory evaluation of bread made from different dough formulations.

#### 3.4.1. Baking Loss and Specific Volume of Bread

**Figure 6:**
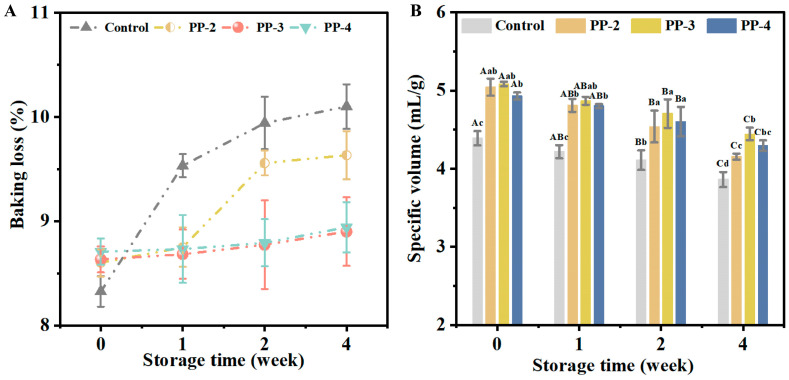
Effect of PP emulsion on the quality attributes of bread. (**A**) Baking loss. (**B**) Specific volume of bread. A statistically significant difference between samples of the same PP emulsion at different storage times is shown by different uppercase letters (*p* < 0.05). A statistically significant difference between samples with various PP emulsions at the same storage time is shown by different lowercase letters (*p* < 0.05).

Baking loss is a key indicator for measuring the baking stability of dough [47]. As shown in Figure 6A, the baking loss increased as the storage period extended. However, doughs containing PP emulsion exhibited significantly lowered baking loss compared with the control group, with PP-3 and PP-4 showing the most stability. Specifically, the increase in baking loss of the PP-3 dough was 84.9% lower than that of the control group after 4 weeks of storage. This reduction in baking loss may result from the redistribution of water in the dough, where PP emulsion increased the bound water content and reduced the free water content, as discussed in Section 3.2.1, whereas the mass loss in baked bread was basically from the loss of water at the surface [48]. This suggested that adding PP emulsion might enhance the water-binding ability of bread, and retain more moisture in the bread, resulting from the relatively complete gluten structure of frozen dough with PP emulsion.

PP emulsion not only reduced baking loss but also increased bread specific volume. Figure 6B demonstrates a general decline in the specific volume after 4 weeks of frozen storage, likely caused by change in water distribution and disruption of the gluten network [49]. Despite this decline, bread made from dough containing PP emulsion maintained higher specific volume values, with the PP-3 dough resulting in a specific volume of 4.44 mL/g after 4 weeks, compared with a decrease from 4.39 to 3.86 mL/g in the control group. This retention of bread volume is likely due to the uniform water distribution observed by LF-NMR and the continuous gluten network observed in the microstructure, both of which contributed to improve retention of gas and water during bread formation [50].

#### 3.4.2. Color Analysis

**Figure A3:**
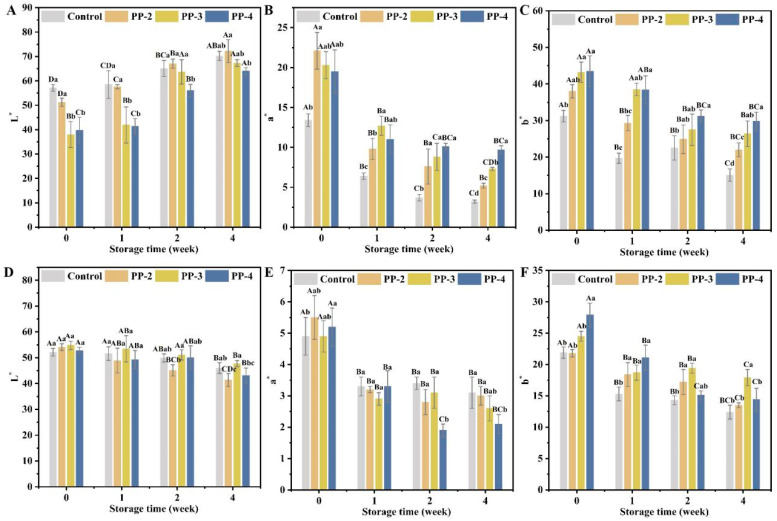
Effect of PP emulsion on the color of bread, (**A**) L*; (**B**) a*; (**C**) b* of crust; (**D**) L*; (**E**) a*; (**F**) b* of crumb. A statistically significant difference between samples of the same PP emulsion at different storage times is shown by different uppercase letters (*p* < 0.05). A statistically significant difference between samples with various PP emulsions at the same storage time is shown by different lowercase letters (*p* < 0.05).

Color intuitively affects consumer perception and preference for baked products and is a key quality indicator. The crust and crumb colors of the bread are shown in Figure A3. Over storage time, all color indicators declined except for L* of the crust. Yang et al. [51] similarly observed contrasting trends in the L* of the crust and the crumb, attributing this to structural differences between the two. In our study, the PP-treated samples retained higher b* values, likely due to the natural yellow-brown hue of PP itself, and the strengthened gluten network provided by PP, which preserved moisture. This retained protein and water promoted the Maillard reaction, especially in the crust. Popov-Raljic et al. [52] found that finer grain structures produce lighter colors, while coarser pores result in darker shades, suggesting that pore wall characteristics may account for the maximum L* value observed in the crumb after four weeks of storage. Additionally, crumb aging typically results in a lighter color. After 4 weeks of storage, the crumb of PP-3 still maintained a darker color (the value of b* maintains 17.9), suggesting that PP-3 formed a robust gluten network structure, which minimized the damage of aging. In conclusion, PP not only reinforced the gluten structure in the frozen environment but also brought a better color performance to the baked bread.

#### 3.4.3. Texture Analysis

##### Porosity of the Bread

**Figure 7:**
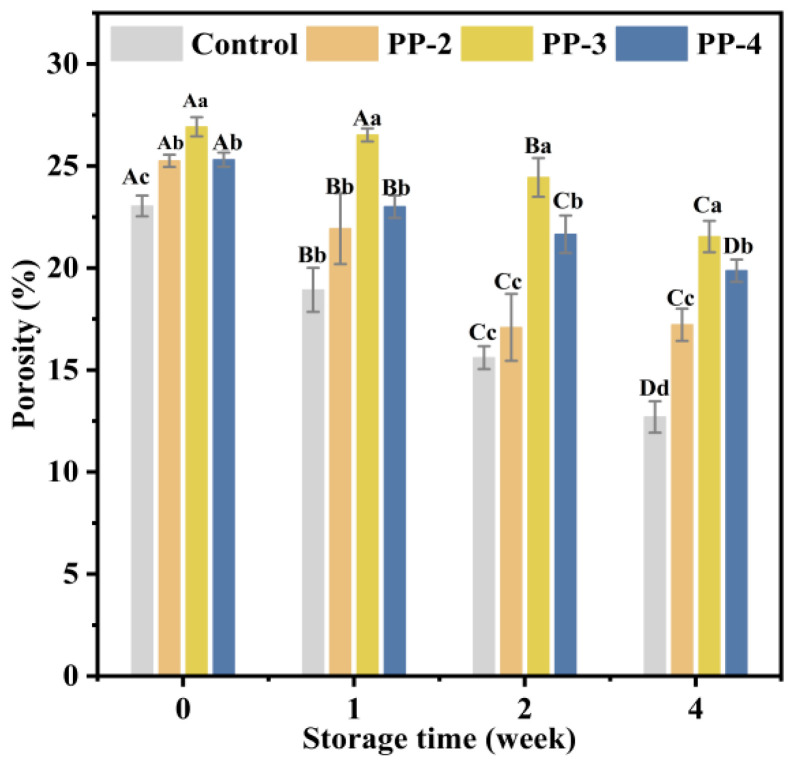
Effect of PP emulsion on the porosity of bread. A statistically significant difference between samples of the same PP emulsion at different storage times is shown by different uppercase letters (*p* < 0.05). A statistically significant difference between samples with various PP emulsions at the same storage time is shown by different lowercase letters (*p* < 0.05).

**Figure A4:**
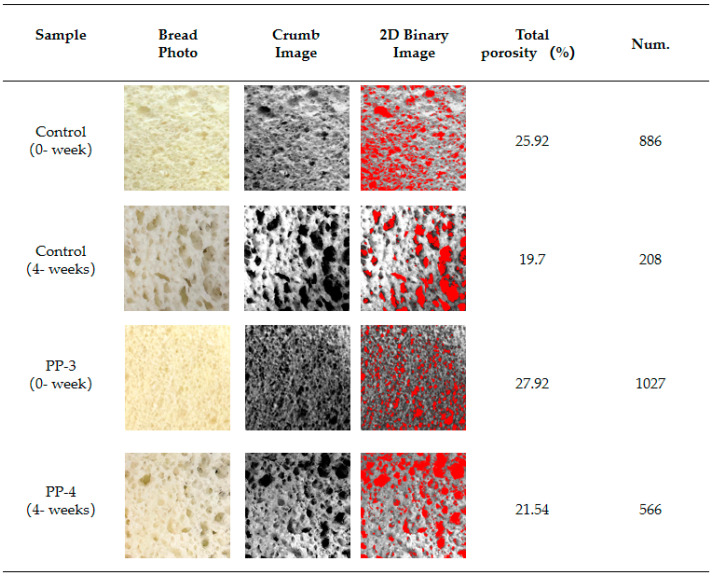
2D and scan plots of the control and PP-3 samples at 0 week and 4 weeks.

As shown in Figure A4, the 2D and scanning images of the control and PP-3 samples at 0 and 4 weeks illustrated the largest and smaller changes in porosity, respectively. As shown in Figure 7, the porosity of all samples generally decreased with increasing storage time, likely due to ice crystal formation, which disrupted the gluten network structure and compromised its ability to retain gas. Although all samples exhibited a decrease in porosity, the bread made from the dough containing PP emulsion maintained a higher porosity, aligning with the trend observed in the specific volume. Specially, the porosity of bread made from the PP-3 dough decreased from 26.92% to 21.54% after 4 weeks, whereas the control group showed a more pronounced reduction of 10.34%, dropping from 23.04%. Moreover, Figure A4 demonstrates that the bread pores of PP-3 were more uniform than those in the control group. This improvement may be due to the PP emulsion’s role in strengthening the gluten network structure, preventing the rupture and bonding of air bubbles. A similar effect of guar and xanthan gum on frozen dough was reported by Hejrani et al. [53]. In conclusion, the addition of the PP emulsifier helped reinforce the network structure of gluten proteins, allowing better gas retention and resulting in smaller, more homogeneously distributed pores.

##### TPA Analysis

**Figure 8:**
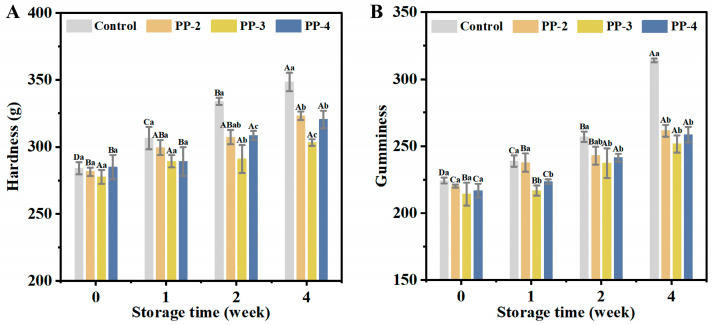

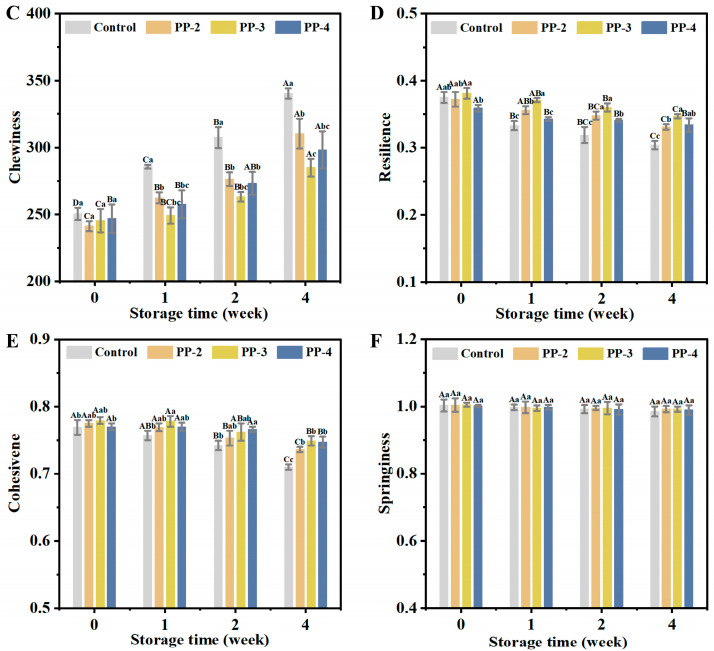
Effect of PP emulsion on bread texture. (**A**) Hardness, (**B**) gumminess, (**C**) chewiness, (**D**) resilience, (**E**) cohesiveness, and (**F**) springiness. A statistically significant difference between samples of the same PP emulsion at different storage times is shown by different uppercase letters (*p* < 0.05). A statistically significant difference between samples with various PP emulsions at the same storage time is shown by different lowercase letters (*p* < 0.05).

Bread hardness is a key indicator of bread quality, often associated with a decrease in resilience over time during storage [54]. The effect of PP emulsion on the texture properties of bread, including hardness, gumminess, chewiness, resilience, cohesiveness, and springiness, is shown in Figure 8. After storing the dough at −18 °C for 4 weeks, a notable decrease in resilience and an increase in hardness of the baked bread were observed. However, when the dough containing PP emulsion was frozen for 4 weeks, the increased hardness in the bread made with PP-3 was 60.2% lower than that of the control group, and the resilience of the bread made with PP-4 was 64.78% higher than that of the control group. This improvement was likely due to the addition of PP emulsion, which reduced the freezable water (FW) content in the frozen dough, thereby preventing the formation of ice crystals and protecting the gluten network (Figure 2) [55]. In addition, the degradation of the gluten structure reduces the gas storage capacity of the dough, impacting the hardness and resilience of the bread [56,57]. Therefore, PP emulsions, with their high freeze–thaw stability, effectively enhanced the quality of baked bread and can be considered a beneficial additive for frozen dough bread making from a freeze–thaw perspective.

##### Crumbliness Analysis

**Figure A5:**
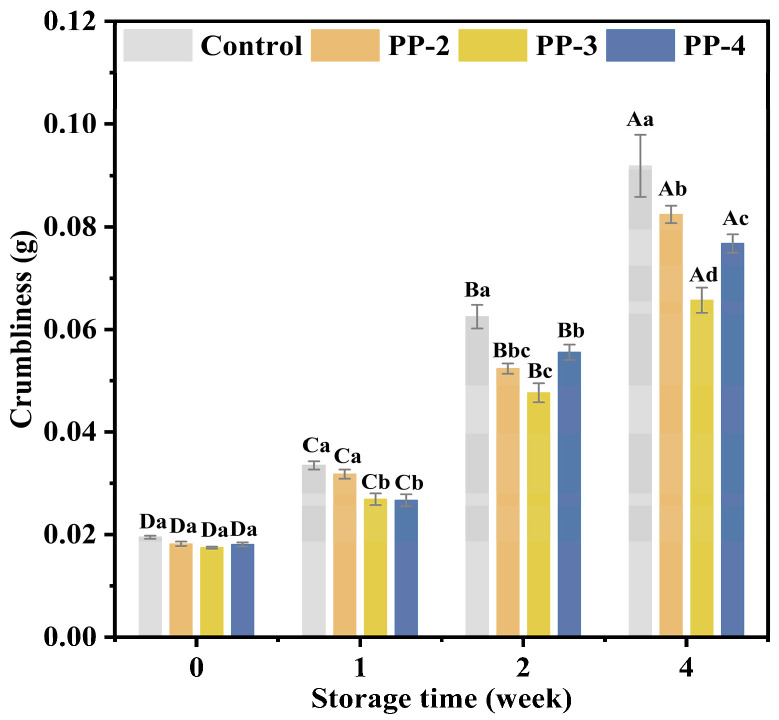
Effect of PP emulsion on crumbliness in dough during frozen storage. A statistically significant difference between samples of the same PP emulsion at different storage times is shown by different uppercase letters (*p* < 0.05). A statistically significant difference between samples with various PP emulsions at the same storage time is shown by different lowercase letters (*p* < 0.05).

As shown in Figure A5, crumbliness of the bread in both the experimental and control groups dramatically increased over time, likely due to increased hardness. After four weeks of freezing, the control group exhibited maximum hardness and minimum cohesiveness, as measured by TPA. In contrast, the PP-3 samples showed only a moderate increase in crumbliness, from 0.017 g to 0.065 g, as PP helped maintain the gluten structure and moisture during freezing, preventing a sharp rise in hardness. This similar trend of increased crumbliness post-freezing was also reported by Purhagen et al. [33], especially a large increase after use of monoglyceride. The results of crumbliness also showed that PP had the ability to act as an effective cryoprotectant.

#### 3.4.4. Sensory Evaluation

**Figure A6:**
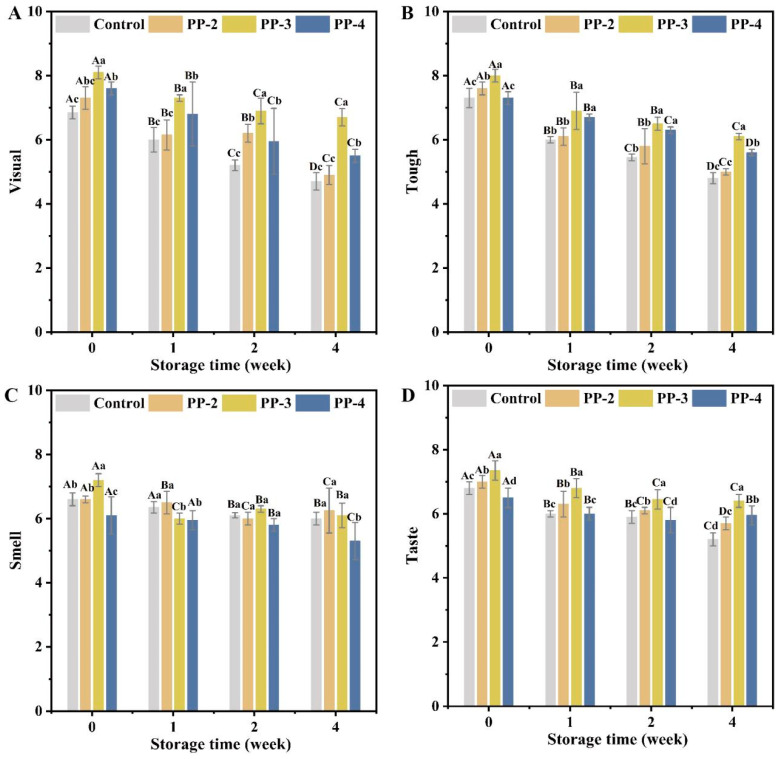
Effect of PP emulsion on sensory evaluation in dough during frozen storage, (**A**) visual; (**B**) tough; (**C**) smell; (**D**) taste A statistically significant difference between samples of the same PP emulsion at different storage times is shown by different uppercase letters (*p* < 0.05). A statistically significant difference between samples with various PP emulsions at the same storage time is shown by different lowercase letters (*p* < 0.05).

Consumer acceptance is crucial for the marketability of food products. Visual perception of bread is largely influenced by color and shape. The inclusion of PP increased the amount of bound water while reducing free water, which helped maintain the gluten structure in the dough, allowing for greater volume retention post-freezing. Moreover, the yellow-brown hue of PP and its contribution to the Maillard reaction facilitated the development of a desirable color. Consequently, bread with PP emulsion received higher visual scores, with PP-3 achieving a stable score of 6.7 after 4 weeks (Figure A6).

The tactile score, reflecting the softness of the bread when pressed, is related to hardness and springiness measured by TPA. Since no significant differences in springiness were observed, variations in tactile scores likely stemmed from the perceived hardness, with PP strengthening the gluten network and enhancing gas retention. However, individual preferences for the beany aroma varied, which was reflected in the aroma and taste scores. Specifically, PP-4 received the lowest aroma score (5.3), as shown in Figure A6. In contrast, PP-3 achieved the higher aroma score, likely due to the Maillard reaction masking the inherent beany odor of PP.

For taste, additional factors beyond aroma appeared to influence scores. After 4 weeks of storage, the experimental group consistently received higher taste scores than the control, even for PP-4. Overall, the sensory evaluation indicated that the addition of PP emulsion enhanced bread quality, especially after extended storage, by stabilizing the gluten structure and retaining more bound water. Furthermore, as PP is a plant-based protein with high biosecurity accessibility, it may hold additional consumer appeal [58].

## 4. Conclusions

In this study, we proposed a strategy utilizing PP emulsion with high freeze-thaw stability as a cryoprotectant for frozen dough. Pea protein offers a cost-efficient solution with substantial economic advantages, while the simplicity of the emulsion preparation and incorporation process makes it highly feasible for practical industrial applications. The results demonstrated that the strong freeze–thaw resistance of PP emulsion significantly improved the quality of frozen dough. The PP emulsion effectively reduced the free water content, inhibited the ice crystal formation, maintained the moisture distribution, and improved the microstructure of dough. Moreover, bread baked with PP emulsion exhibited reduced baking losses and retained texture and sensory characteristics, even after 4 weeks of freezing. Notably, PP-3 displayed more appealing flavor and color. Thus, this study underscores the potential of PP emulsions as an efficient cryoprotectant for dough and offers valuable insights into the frozen storage of dough. Nevertheless, the effects of PP emulsion on the microbial stability of frozen dough during freezing and its impact on the quality of dough with longer freezing storage warrant further investigation in future studies. 

## Figures and Tables

**Table 1:** Formulations of different PP emulsions (Control, 2% PP, 3% PP, and 4% PP).

Group	Water (g)	Butter (g)	PP (g)
Control	65	5	0
PP-2	65	5	1.4
PP-3	65	5	2.1
PP-4	65	5	2.8

**Table 2:** Effect of PP emulsion on the moisture mobility of frozen dough.

Samples	PT_21_/%	PT_22_/%	PT_23_/%
0-week-Control	14.71 ± 0.28 ^Aa^	81.05 ± 0.18 ^Bab^	4.24 ± 0.15 ^Ba^
0-week-PP-2	14.65 ± 0.17 ^Aa^	81.16 ± 0.15 ^Ba^	4.19 ± 0.24 ^Ba^
0-week-PP-3	14.90 ± 0.09 ^Aa^	80.80 ± 0.11 ^Bb^	4.30 ± 0.19 ^Aa^
0-week-PP-4	14.62 ± 0.25 ^Aa^	81.09 ± 0.22 ^Aab^	4.29 ± 0.22 ^Aa^
4-week-Control	12.86 ± 0.09 ^Bc^	83.35 ± 0.10 ^Aa^	5.78 ± 0.08 ^Aa^
4-week-PP-2	13.85 ± 0.01 ^Bb^	81.46 ± 0.05 ^Ab^	4.69 ± 0.06 ^Ab^
4-week-PP-3	14.29 ± 0.23 ^Ba^	81.23 ± 0.30 ^Ab^	4.48 ± 0.14 ^Ab^
4-week-PP-4	14.31 ± 0.08 ^Aa^	81.12 ± 0.31 ^Ab^	4.57 ± 0.23 ^Ab^

A statistically significant difference between samples of the same PP emulsion at different storage times is shown by different uppercase letters (*p* < 0.05). A statistically significant difference between samples with various PP emulsions at the same storage time is shown by different lowercase letters (*p* < 0.05).

## Data Availability

The data used to support the findings of this study can be made available by the corresponding author upon request.

## References

[1] Qian M., Liu D., Zhang X., Yin Z., Ismail B.B., Ye X., Guo M. (2021). A Review of Active Packaging in Bakery Products: Applications and Future Trends. Trends Food Sci. Technol..

[2] Yang Z., Jin Y., Xu X. (2024). Mechanism of Frozen Dough Deterioration and the Behavior of Different Wheat Starch Types during Storage. J. Cereal Sci..

[3] Phimolsiripol Y., Siripatrawan U., Cleland D.J. (2011). Weight Loss of Frozen Bread Dough under Isothermal and Fluctuating Temperature Storage Conditions. J. Food Eng..

[4] Zhang T., Teng Y., He Y., Li Y., Yuan Y., Li B., Chen Y., Zhu X. (2024). Elucidate the molecular basis of ampholytic chitosan as a high-performance cryoprotectant to myosin denaturation: The importance of saccharide charges. Food Hydrocoll..

[5] Wang H., Fan H., Zhang S., Xia C., Wang J., Zhang Y., Liu T. (2023). Effects of Tremella Polysaccharide on Frost Resistance of Frozen Dough Considering Water State, Physical Property and Gluten Structure. LWT.

[6] Zhu X., Yuan P., Zhang T., Wang Z., Cai D., Chen X., Shen Y., Xu J., Song C., Goff D. (2022). Effect of carboxymethyl chitosan on the storage stability of frozen dough: State of water, protein structures and quality attributes. Food Res. Int..

[7] Zhu X., Chen Y., Zhang N., Luo Y., Peng R., Chen L., Xu J., Teng Y., Li B., Ding W. (2024). Chickpea Peptide as a Plant-Based Cryoprotectant in Frozen Dough: Insight into the Water States, Gluten Structures, and Storage Stabilities. LWT.

[8] Chen N., Yang Q., Zhang C.C., Chen H.Q. (2023). Impact of Basil Seed Gum on the Textural, Rheological Properties, Water State, Gluten Depolymerization and Microstructure of Frozen Dough. J. Cereal Sci..

[9] Mattice K.D., Marangoni A.G. (2018). Gelatinized Wheat Starch Influences Crystallization Behaviour and Structure of Roll-in Shortenings in Laminated Bakery Products. Food Chem..

[10] Ma S., Han W. (2019). Application in Bakery Products. Dietary Fiber: Properties, Recovery, and Applications.

[11] Zhang H., Fan H., Xu X., Xu D. (2024). Deterioration Mechanisms and Quality Improvement Methods in Frozen Dough: An Updated Review. Trends Food Sci. Technol..

[12] Shu G., Khalid N., Zhao Y., Neves M.A., Kobayashi I., Nakajima M. (2016). Formulation and Stability Assessment of Ergocalciferol Loaded Oil-in-Water Nanoemulsions: Insights of Emulsifiers Effect on Stabilization Mechanism. Food Res. Int..

[13] Bu N., Huang L., Cao G., Pang J., Mu R. (2022). Stable O/W Emulsions and Oleogels with Amphiphilic Konjac Glucomannan Network: Preparation, Characterization, and Application. J. Sci. Food Agric..

[14] Sanz T., Laguna L., Salvador A. (2015). Biscuit Dough Structural Changes During Heating: Influence of Shortening and Cellulose Ether Emulsions. LWT.

[15] Ishibashi C., Hondoh H., Ueno S. (2016). Influence of Morphology and Polymorphic Transformation of Fat Crystals on the Freeze-Thaw Stability of Mayonnaise-Type Oil-in-Water Emulsions. Food Res. Int..

[16] Ding X., Li T., Zhang H., Guan C., Qian J., Zhou X. (2020). Effect of barley antifreeze protein on dough and bread during freezing and freeze-thaw cycles. Foods.

[17] Chen J., Chen X., Zhu Q., Chen F., Zhao X., Ao Q. (2013). Determination of the domain structure of the 7S and 11S globulins from soy proteins by XRD and FTIR: Determination of the domain structure of soy globulins. J. Sci. Food Agric..

[18] Boukid F. (2022). The Realm of Plant Proteins with Focus on Their Application in Developing New Bakery Products. Adv. Food Nutr. Res..

[19] Teng Y., Zhang T., Dai H., Wang Y., Xu J., Zeng X., Li B., Zhu X. (2023). Inducing the structural interplay of binary pulse protein complex to stimulate the solubilization of chickpea (*Cicer arietinum* L.) protein isolate. Food Chem..

[20] Nishinari K., Fang Y., Nagano T., Guo S., Wang R. (2017). Soy as a Food Ingredient. Proteins in Food Processing.

[21] Zhang X., Chen Y., Li R., Shi Y., Zhao Y., Li B., Chen Y., Zhu X. (2024). Fabrication of Pea Protein Isolate-Stabilized Oil-in-Water Emulsions with High Freeze-Thaw Stability: Effect of High Intensity Ultrasonic on Emulsions and Interfacial Protein Structure. Food Hydrocoll..

[22] Tong H., Wang J., Qi L., Gao Q. (2023). Starch-Based Janus Particle: Fabrication, Characterization and Interfacial Properties in Stabilizing Pickering Emulsion. Carbohydr. Polym..

[23] Baier-Schenk A., Handschin S., Conde-Petit B. (2005). Ice in Prefermented Frozen Bread Dough—An Investigation Based on Calorimetry and Microscopy. Cereal Chem..

[24] He Y., Guo J., Ren G., Cui G., Han S., Liu J. (2020). Effects of Konjac Glucomannan on the Water Distribution of Frozen Dough and Corresponding Steamed Bread Quality. Food Chem..

[25] Feng Y., Mu T., Zhang M., Ma M. (2020). Effects of Different Polysaccharides and Proteins on Dough Rheological Properties, Texture, Structure and in Vitro Starch Digestibility of Wet Sweet Potato Vermicelli. Int. J. Biol. Macromol..

[26] Ellis H.R. (2022). On “Tissue Sulfhydryl Groups” by George L. Ellman. Arch. Biochem. Biophys..

[27] Chen X., Su T., Yang H., Lei H., Meng M., Luo X., Ou C., Jia L., Sang S. (2023). Effect of Fish Skin Gelatin on Characteristics and Staling Properties of Bread Made from Pre-Baked Frozen Dough. Food Biosci..

[28] Correa M.J., Pérez G.T., Ferrero C. (2012). Pectins as Breadmaking Additives: Effect on Dough Rheology and Bread Quality. Food Bioprocess Technol..

[29] Gao J., Wong J.X., Lim J.C.S., Henry J., Zhou W. (2015). Influence of bread structure on human oral processing. J. Food Eng..

[30] Peng B., Li Y., Ding S., Yang J. (2017). Characterization of Textural, Rheological, Thermal, Microstructural, and Water Mobility in Wheat Flour Dough and Bread Affected by Trehalose. Food Chem..

[31] Beltrão Martins R., Nunes M.C., Ferreira L.M.M., Peres J.A., RNA Barros A.I., Raymundo A. (2020). Impact of acorn flour on gluten-free dough rheology properties. Foods.

[32] Lu L., Yang Z., Guo X.N., Xing J.J., Zhu K.X. (2021). Effect of NaHCO_3_ and Freeze–Thaw Cycles on Frozen Dough: From Water State, Gluten Polymerization and Microstructure. Food Chem..

[33] Purhagen J.K., Sjöö M.E., Eliasson A.C. (2011). Starch affecting anti-staling agents and their function in freestanding and pan-baked bread. Food Hydrocoll..

[34] Cacak-Pietrzak G., Dziki D., Gawlik-Dziki U., Parol-Nadłonek N., Kalisz S., Krajewska A., Stępniewska S. (2023). Wheat Bread Enriched with Black Chokeberry (*Aronia melanocarpa* L.) Pomace: Physicochemical Properties and Sensory Evaluation. Appl. Sci..

[35] Xu W., Jia Y., Li J., Sun H., Cai L., Wu G., Kang M., Zang J., Luo D. (2024). Pickering Emulsion with High Freeze-Thaw Stability Stabilized by Xanthan Gum/Lysozyme Nanoparticles and Konjac Glucomannan. Int. J. Biol. Macromol..

[36] Liu M., Liang Y., Zhang H., Wu G., Wang L., Qian H., Qi X. (2018). Comparative Study on the Cryoprotective Effects of Three Recombinant Antifreeze Proteins from Pichia Pastoris GS115 on Hydrated Gluten Proteins during Freezing. J. Agric. Food Chem..

[37] Matuda T.G., Parra D.F., Lugão A.B., Tadini C.C. (2005). Influence of vegetable shortening and emulsifiers on the unfrozen water content and textural properties of frozen French bread dough. LWT-Food Scicen Technol..

[38] Liang Y., Qu Z., Liu M., Zhu M., Zhang X., Wang L., Jia F., Zhan X., Wang J. (2021). Further Interpretation of the Strengthening Effect of Curdlan on Frozen Cooked Noodles Quality during Frozen Storage: Studies on Water State and Properties. Food Chem..

[39] Liu S., Gu S., Shi Y., Chen Q. (2024). Alleviative Effects of Mannosylerythritol Lipid-A on the Deterioration of Internal Structure and Quality in Frozen Dough and Corresponding Steamed Bread. Food Chem..

[40] Wang Z., Ma S., Li L., Huang J. (2022). Synergistic Fermentation of Lactobacillus Plantarum and Saccharomyces Cerevisiae to Improve the Quality of Wheat Bran Dietary Fiber-Steamed Bread. Food Chem. X.

[41] Zhang J., Li J., Fan L. (2024). Effect of Starch Granule Size on the Properties of Dough and the Oil Absorption of Fried Potato Crisps. Int. J. Biol. Macromol..

[42] Jeong S., Park Y., Lee S. (2021). Assessment of Turanose as a Sugar Alternative in a Frozen Dough System: Rheology, Tomography, and Baking Performance. LWT.

[43] Tu Y., Zhang X., Wang L. (2023). Salt Ions Induced Rice Bran Protein Nanoparticle Stabilized High Internal Phase Emulsion as a Fat Substitute on Breads. J. Cereal Sci..

[44] Lee J.Y., Tiffany C.R., Mahan S.P., Kellom M., Rogers A.W.L., Nguyen H., Stevens E.T., Masson H.L.P., Yamazaki K., Marco M.L. (2024). High Fat Intake Sustains Sorbitol Intolerance after Antibiotic-Mediated Clostridia Depletion from the Gut Microbiota. Cell.

[45] Zhao B., Hou L., Liu T., Liu X., Fu S., Li H. (2023). Insight into Curdlan Alleviating Quality Deterioration of Frozen Dough during Storage: Fermentation Properties, Water State and Gluten Structure. Food Chem. X.

[46] Yang S., Zhao X., Liu T., Cai Y., Deng X., Zhao M., Zhao Q. (2024). Effects of Apple Fiber on the Physicochemical Properties and Baking Quality of Frozen Dough during Frozen Storage. Food Chem..

[47] Wang P., Chen H., Mohanad B., Xu L., Ning Y., Xu J., Wu F., Yang N., Jin Z., Xu X. (2014). Effect of Frozen Storage on Physico-Chemistry of Wheat Gluten Proteins: Studies on Gluten-, Glutenin- and Gliadin-Rich Fractions. Food Hydrocoll..

[48] Zhao F., Li Y., Li C., Ban X., Cheng L., Hong Y., Gu Z., Li Z. (2022). Insight into the Regulations of Rice Protein on the Gluten-Free Bread Matrix Properties. Food Hydrocoll..

[49] Tiwari U., Cummins E., Sullivan P., Flaherty J.O., Brunton N., Gallagher E. (2011). Probabilistic Methodology for Assessing Changes in the Level and Molecular Weight of Barley β-Glucan during Bread Baking. Food Chem..

[50] Zhang H., Wei A., Zhou S., Zhang H., Xia N., Wang J., Ma Y., Fan M. (2024). Effect of the Substitution of Butter by Double Cross-Linked Egg Yolk Granules/Sodium Alginate Emulsion Gel on Properties of Baking Dough during Frozen Storage. Food Chem..

[51] Yang Z., Xu D., Zhou H., Wu F., Xu X. (2022). New Insight into the Contribution of Wheat Starch and Gluten to Frozen Dough Bread Quality. Food Biosci..

[52] Popov-Raljic J.V., Mastilovic J.S., Lalicic-Petronijevic J.G., Popov V.S. (2009). Investigations of bread production with postponed staling applying instrumental measurements of bread crumb color. Sensors.

[53] Hejrani T., Sheikholeslami Z., Mortazavi A., Davoodi M.G. (2017). The properties of part baked frozen bread with guar and xanthan gums. Food Hydrocoll..

[54] Dong Y.N., Karboune S. (2021). A Review of Bread Qualities and Current Strategies for Bread Bioprotection: Flavor, Sensory, Rheological, and Textural Attributes. Compr. Rev. Food Sci. Food Saf..

[55] Mao X., Liu Z., Ma J., Pang H., Zhang F. (2011). Characterization of a Novel β-Helix Antifreeze Protein from the Desert Beetle Anatolica Polita. Cryobiology.

[56] Chen J., Xiao J., Tu J., Yu L., Niu L. (2023). The Alleviative Effect of Sweet Potato Protein Hydrolysates on the Quality Deterioration of Frozen Dough Bread in Comparison to Trehalose. LWT.

[57] Zhou B., Dai Y., Guo D., Zhang J., Liang H., Li B., Sun J., Wu J. (2022). Effect of Desalted Egg White and Gelatin Mixture System on Frozen Dough. Food Hydrocoll..

[58] Shen Y., Babu K.S., Amamcharla J., Li Y. (2020). Emulsifying properties of pea protein/guar gum conjugates and mayonnaise application. Int. J. Food Sci. Technol..

